# Slimmer’s Palsy Following Weight Loss Associated With Metastatic Breast Cancer: A Case Report

**DOI:** 10.7759/cureus.52519

**Published:** 2024-01-18

**Authors:** Reid W Collis, Alaric J Gee, Patrick Dillon, Michael Warwick

**Affiliations:** 1 Physical Medicine and Rehabilitation, University of Virginia, Charlottesville, USA; 2 Hematology and Oncology, University of Virginia, Charlottesville, USA

**Keywords:** common, neuropathy, peroneal, cancer, breast, report, case, malignancy, palsy, slimmer's

## Abstract

Common peroneal neuropathy (CPN), also known as Slimmer’s Palsy, is an isolated peripheral neuropathy typically associated with rapid weight loss resulting in loss of adipose tissue and subsequent nerve compression at the fibular head and is up to three times more common in individuals with malignancy. In this case report, we describe the diagnosis of CPN in a 54-year-old female with a 2.5-month history of atraumatic left foot drop and left ankle paresthesias, preceded by a 35-40 pound weight loss over the prior 3.5 month period in the setting of metastatic breast cancer.

## Introduction

Common peroneal neuropathy (CPN) is a functionally debilitating mononeuropathy affecting the deep and superficial peroneal nerve branches and is often considered the most common mononeuropathy of the lower extremities [1]. CPN is up to three times more common in individuals with malignancy than in individuals without malignancy [2]. Common presenting symptoms of CPN include foot drop due to ankle dorsiflexion and eversion weakness, with inversion of the ankle not affected [3]. This case report details the evaluation of a 54-year-old female with metastatic breast cancer who presented with a 2.5-month history of atraumatic, insidious onset left-sided foot drop. Any patient presenting with new foot drop warrants a prompt and thorough physical examination and diagnostic evaluation for both common lower motor neuron conditions such as radiculopathy and CPN and less common but potentially emergent conditions such as acute stroke or compressive myelopathy [4,5]. This case further highlights the importance of maintaining a broad differential diagnosis while maintaining a high index of suspicion for weight loss as a potential causative factor within this patient population.

## Case presentation

The patient was a 54-year-old female with a past medical history of metastatic triple-positive breast cancer (estrogen receptor-positive, progesterone receptor-positive, and HER2 receptor-positive). The breast cancer had an activating mutation of PIK3CA, but no other reported mutations. The patient developed the complication of malignant ascites as her only site of disease recurrence exactly nine years after an initial mastectomy. Tissue from a confirmatory omental biopsy found that the recurrent breast cancer no longer overexpressed HER2 but did remain positive for estrogen receptor and progesterone receptor. For the metastatic disease in the peritoneum/omentum, she received three lines of cytotoxic and/or targeted therapy (palbociclib/fulvestrant, alpelisib/fulvestrant, and nab-paclitaxel). The patient started nab-paclitaxel two months prior to presenting to our outpatient electrodiagnostic clinic. 

The patient subsequently presented to the clinic with a 2.5-month history of left ankle dorsiflexion weakness and foot drop, which began without trauma, change in physical activity such as leg crossing or kneeling on hard surfaces, or other precipitating events. She initially noted a tingling sensation of the left foot and ankle followed by subjective swelling and cold sensation relative to the right side. Upon further questioning, she endorsed unintentional weight loss of 35-40 lb (16-18 kg) over the past six months, citing intermittent nausea and poor appetite. Of note, she was unable to continue working as a house cleaner due to a left foot drop.

A review of the electronic medical record revealed a recent body weight of 60.3 kg or 132.9 lb and a BMI of 22.1 kg/m^2^. Previously documented weight at an oncology appointment approximately six months prior was noted to be 74.8 kg or 164.9 lb, a difference of 14.5 kg or 32 lbs. Physical examination was notable for 5/5 strength with right ankle dorsiflexion, plantarflexion, inversion, eversion, and great toe extension; 0/5 strength with left ankle dorsiflexion, eversion, and great toe extension; and 5/5 strength with left ankle plantarflexion, inversion as well as bilateral knee extension and hip flexion. The sensation was also diminished to light touch along the dorsal left foot. 

On review of diagnostic imaging studies, an MRI of the lumbar spine one month prior demonstrated mild multilevel spinal degenerative changes with scattered neuroforaminal narrowing, most pronounced on the left at L4-L5, which was moderate, and no evidence of osseous or perineural metastatic disease. Three-view left foot x-ray from three weeks prior was also found to be without osseous abnormality, malalignment, or significant degenerative changes. Notably, the patient had no other neuropathy symptoms related to the nab-paclitaxel, and she had no pre-existing neuropathy nor paraneoplastic/vasculitic syndromes prior to her acute presentation.

Nerve conduction studies revealed focal left deep peroneal motor conduction slowing and partial motor conduction block across the fibular head recording at both the tibialis anterior and extensor digitorum brevis (Table 1).

**Table 1:** Lower Limb Nerve Conduction Study Results Lower limb motor nerve conduction study results demonstrating unilateral entrapment neuropathy at the level of the fibular head, characterized by focal deep fibular (peroneal) MCVs slowing and partial motor conduction block. Left superficial fibular (peroneal) sensory nerve action potentials are also mildly reduced MCVs, motor conduction velocities

	Latency		Amplitude		Segment	Distance	Conduction velocity	
Site	(ms)	Normal	(mV)	Normal		(mm)	(m/s)	Normal
Fibular Head	3.6		1.11	-				
Popliteal Fossa	8.9		0.55	-	Popliteal Fossa-Fibular Head	110	21	-
Fibular Head	4.0		0.99	-				
Ankle	5.4		2.5	>1.10				
Below Fibular Head	13.7	-	2.2	-	Below Fibular Head-Ankle	330	40	>39
Popliteal Fossa	17.1	-	0.35	-	Popliteal Fossa-Below Fibular Head	90	26	>42
Ankle	3.7		3.4	>1.10				
Ankle	5.5		5.4	>5.3				
Knee	15.1	-	4.8	-	Knee-Ankle	360	38	>37

	Latency (Peak)		Negative Peak - Start Amplitude		Segment	Distance
Site	(ms)	Normal	µV	Normal		(mm)
Left Superficial Fibular Sensory						
Leg-Ankle	2.3	-	4	>6	Leg-Ankle	90
Right Superficial Fibular Sensory						
Leg-Ankle	3.2	-	7	>6	Leg-Ankle	120
Left Sural Sensory						
Calf-Lateral Malleolus	3.4		7	>4	Calf-Lateral Malleolus	120
Right Sural Sensory						
Calf-Lateral Malleolus	3.3		8	>4	Calf-Lateral Malleolus	120

Left superficial peroneal sensory nerve action potentials were mildly reduced relative to the right side. Left deep peroneal compound muscle action potential (CMAP) amplitudes were mildly reduced at the tibialis anterior bilaterally but grossly symmetric and were within normal limits at the extensor digitorum brevis. Needle EMG examination was notable for fibrillation potentials and positive sharp waves in the left tibialis anterior, peroneus longus, and extensor hallucis longus, indicative of active, ongoing denervation (Table 2).

**Table 2:** Lower Limb EMG Results Lower limb EMG results demonstrating active denervation in the tibialis anterior, fibularis (peroneus) longus, and extensor hallucis longus, absent volitional motor activity in the tibialis anterior or extensor hallucis longus, and markedly reduced motor unit recruitment in the fibularis (peroneus) longus. nml, normal; EMG, electromyography

Side	Muscle	Nerve	Root	Insertional Activity	Fibrillations	Positive Sharp Waves	Amplitude	Durration	Polyphasic	Recruitment	Comment
Right	Tibialis Posterior	Tibial	L5-S1	Nml	Nml	Nml	Nml	Nml	0	Nml	
Left	Tibialis Anterior	Fibular, Deep Fibular	L4-L5	Nml	3+	3+					No volitional activity
Left	Gastrocnemius Medial Head	Tibial	S1-S2	Nml	Nml	Nml	Nml	Nml	0	Nml	
Left	Tibialis Posterior	Tibial	L5-S1	Nml	Nml	Nml	Nml	Nml	0	Nml	
Left	Vastus Medialis	Femoral	L2-L4	Nml	Nml	Nml	Nml	Nml	0	Nml	
Left	Extensor Hallucis Longus	Fibular, Deep Fibular	L5-S1	Nml	3+	3+					No volitional activity
Left	Fibularis Longus	Fibular, Superficial	L5-S1	Nml	2+	2+	Nml	Nml	1+	Reduced	Single motor unit
Left	Biceps Femoris (Short Head)	Sciatic	L5-S1	Nml	Nml	Nml	Nml	Nml	0	Nml	
Left	Tensor Fascia Lata	Superior Gluteal	L4-S1	Nml	Nml	Nml	Nml	Nml	0	Nml	
Left	Lumbar Paraspinals	Rami	L3-S1	Nml	Nml	Nml					

The extensor hallucis longus and tibialis anterior demonstrated no volitional motor activity, suggestive of complete functional denervation, while the peroneus longus demonstrated a markedly reduced, single motor unit recruitment pattern, suggestive of near complete functional denervation. There was no evidence of tibial or sciatic neuropathy, lumbosacral radiculopathy, or generalized peripheral polyneuropathy. 

Following a discussion of the test results and CPN diagnosis, the patient was placed in an ankle-foot-orthosis (AFO) brace and managed conservatively without decompressive surgery. At a four-month follow-up, she regained approximately 80% of her functional ability, graduated out of wearing the AFO brace, and was able to return to work cleaning houses. She had also regained 2.8 kg or 6.4 lb from a nadir of 60.3 kg or 132.9 lb over this period.

## Discussion

**Figure 1:**
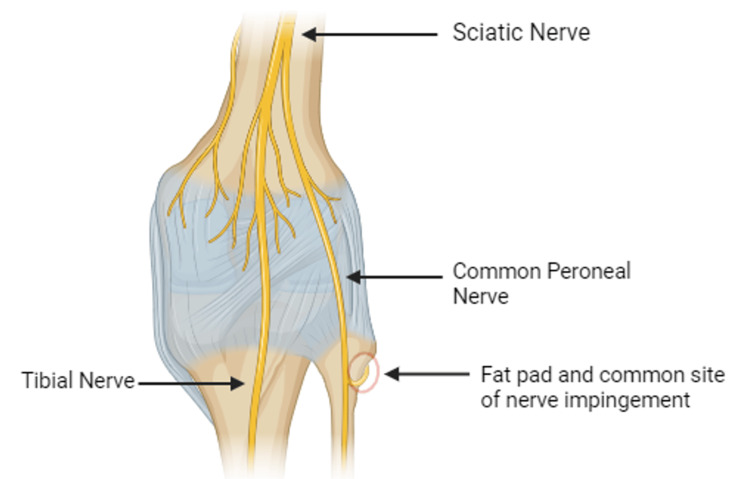
Posterior View of the Knee Demonstrates the major nerves passing through the posterior knee and the common site of peroneal nerve impingement. Created using BioRender and published with permission.

CPN is an isolated mononeuropathy affecting both the deep and superficial peroneal nerve branches at the level of the knee, most often caused by compression of the peroneal nerve as it passes over the fibular head. This syndrome is typically characterized by foot drop secondary to ankle dorsiflexion and eversion weakness with inversion being spared [3]. Though sensory symptoms are present in most patients, the impact is often comparatively minor [3]. At the level of the fibular head, the common peroneal nerve travels quite superficially, covered only by skin and a subcutaneous fat pad, rendering the common peroneal nerve particularly susceptible to compression [6] (Figure 1).

The occurrence of CPN is higher in individuals with malignancy than in the general population [2]. The difference in relative rates of CPN between these groups is often attributed to mechanical compression of the peroneal nerve secondary to weight loss and loss of the fat pad, which may occur due to primary disease effects and/or secondarily to treatment such as chemotherapy. This phenomenon is additionally well-documented among the general patient population and is often described after rapid weight loss such as postgastric bypass surgery or with extreme dieting. Though less common, other mechanisms of CPN among patients with malignancy have also been reported including paraneoplastic vasculitis, local infiltrative metastatic lesions such as neurofibromas, bony metastasis to the lumbar spine involving the L5 nerve root, and chemotherapy-induced peripheral neuropathy [7-10]. More recently, studies have highlighted a preventative role for supplemental nutrition, demonstrating a 62% lower incidence of CPN in individuals with malignancy receiving adequate nutritional support compared to individuals without [11].

Electrodiagnostic features of CPN can include focal demyelination, sensorimotor axonal loss, or both [12]. Denervation is more common and severe in muscles supplied by the deep peroneal nerve branch including the tibialis anterior, extensor digitorum longus, extensor hallucis longus, and extensor digitorum brevis, with focal demyelination and axonal loss occurring at roughly the same frequency [13]. While electrodiagnostic studies are considered the standard criterion for diagnosing CPN secondary to mechanical compression, additional diagnostic workup may aid in the diagnosis of less common causes of CPN, including imaging and laboratory testing such as lumbar spine MRI, MR neurography, metabolic and systemic disease screening tests such as fasting blood glucose, thyroid function tests, vitamin B12, folate, and thiamine levels, inflammatory markers, and connective tissue screening labs including ANA, complement levels, and anti-double-stranded DNA.

In this patient presenting with atraumatic, insidious onset left foot drop and no concomitant signs or symptoms indicative of local or diffuse metastasis or vasculitis, history of significant weight loss (approximately 15 kg over six months prior to electrodiagnostic testing) in the setting of metastatic malignancy suggests mechanical CPN entrapment at the fibular head as the most likely etiology. The absence of tibial or sciatic neuropathy, lumbosacral radiculopathy, or generalized peripheral polyneuropathy further suggests CPN as the most likely diagnosis. Given the mixed prognostic picture, with present recordable CMAPs at the tibialis anterior and extensor digitorum brevis (positive prognostic factor) but absent motor unit recruitment in the tibialis anterior (negative prognostic factor), the patient was counseled that while some spontaneous recovery of function is anticipated, with observed recovery rates reported between 78% and 90%, full spontaneous recovery of function is far from guaranteed [14-16].

Ultimately, this patient elected to proceed with conservative management as previously noted including the use of an AFO, reporting significant ongoing improvement at a four-month follow-up. Additional supportive measures can include activity modification, focused physical therapy, and weight loss mitigation efforts. For more severe cases including complete lesions or those with progressive neurologic deficit, early surgical intervention including CPN decompression and/or tibialis posterior tendon transfer may be indicated to maintain and/or improve function.

## Conclusions

In patients with CPN and concomitant solid malignancy, physical entrapment of the peroneal nerve at the level of the fibular head remains the most likely etiology, particularly in patients who have experienced significant weight loss. Other less commonly reported causes of CPN include vasculitis, metastasis, and chemotherapy-induced peripheral neuropathy. Clinicians should maintain a high index of suspicion for “Slimmer’s palsy” while maintaining a broad differential with diagnostic testing including EMG, imaging, and labs aiding in the diagnosis while ruling out other causes. While the degree of symptoms may vary, CPN can be functionally debilitating and detrimental to one’s mobility and activities of daily living in addition to vocational and recreational pursuits. Patients should be counseled that in most cases, significant recovery of strength and sensation is common, particularly in individuals where cessation of further weight loss can be achieved. Furthermore, adequate nutritional support may provide an important preventative role in the development of CPN. For those with poor prognostic findings on EMG or progressive neurologic deficits, however, early surgical decompression may be warranted.
